# Aggregate of nanoparticles: rheological and mechanical properties

**DOI:** 10.1186/1556-276X-6-114

**Published:** 2011-02-03

**Authors:** Yu Wang, Xiaojun Wu, Wei Yang, Yuanming Zhai, Banghu Xie, Mingbo Yang

**Affiliations:** 1 College of Polymer Science and Engineering, State Key Laboratory of Polymer Materials Engineering, Sichuan University, Chengdu, 610065, China

## Abstract

The understanding of the rheological and mechanical properties of nanoparticle aggregates is important for the application of nanofillers in nanocompoistes. In this work, we report a rheological study on the rheological and mechanical properties of nano-silica agglomerates in the form of gel network mainly constructed by hydrogen bonds. The elastic model for rubber is modified to analyze the elastic behavior of the agglomerates. By this modified elastic model, the size of the network mesh can be estimated by the elastic modulus of the network which can be easily obtained by rheology. The stress to destroy the aggregates, i.e., the yield stress (*σ_y_*), and the elastic modulus (*G'*) of the network are found to be depended on the concentration of nano-silica (*ϕ*, wt.%) with the power of 4.02 and 3.83, respectively. Via this concentration dependent behavior, we can extrapolate two important mechanical parameters for the agglomerates in a dense packing state (*ϕ *= 1): the shear modulus and the yield stress. Under large deformation (continuous shear flow), the network structure of the aggregates will experience destruction and reconstruction, which gives rise to fluctuations in the viscosity and a shear-thinning behavior.

## Introduction

An important application of nano-fillers is to construct nanocomposites with high performance of mechanical properties or certain functionality [1]. Usually, for their high surface energy, nano-fillers exist in the form of agglomerates. Interestingly, some agglomerates, such as nano-silica and nano-titanium dioxide, can present a chain-like form what is called nanoparticle chain aggregates (NCA) and its dynamic properties have been mainly revealed by the work of Friedlander, Bandyopadhyaya and Rong et. al [2-8]. The ductility of NCA is believed to be related to the sliding/rotation of the primary nanoparticles and the elasticity comes from the effect of the surface energy of nanoparticles [3,5,6]. This deformation and elasticity behaviors are very similar to polymer chains as the flexibility of a polymer chain is generated by the rotation of the backbone bonds and the elasticity is driven by the principle of entropy increase.

In fact, the nano- or micro-mechanical properties of the agglomerate are one of the fundamental issues to understand not only the mechanical or the melt rheological properties of nanocomposites, but also the process of the dispersion. However, the work on this area is still seldom reported [7,9,10]. For common nanoparticles, the elemental force between the nanoparticles is the Van der Waals force, however, for the nanoparticles with polar groups, such as fumed nano-silica, there is another stronger interaction, hydrogen bonds, owing to the silanol (Si-OH) on the nanoparticle surface [11]. Fumed nano-silica has been widely used as a modifier for rheological properties of coatings [12] or a reinforcement/functionalization filler in polymer based nanocomposites [13-15]. Certainly, the existence of the hydrogen bonds will affect the dispersion, the nano- or micro-mechanical properties of the nanoparticle agglomerate and finally, the application of nano-silica [15,16].

## Experiment

### Materials, sample preparation and characterizations

**Figure 1:**
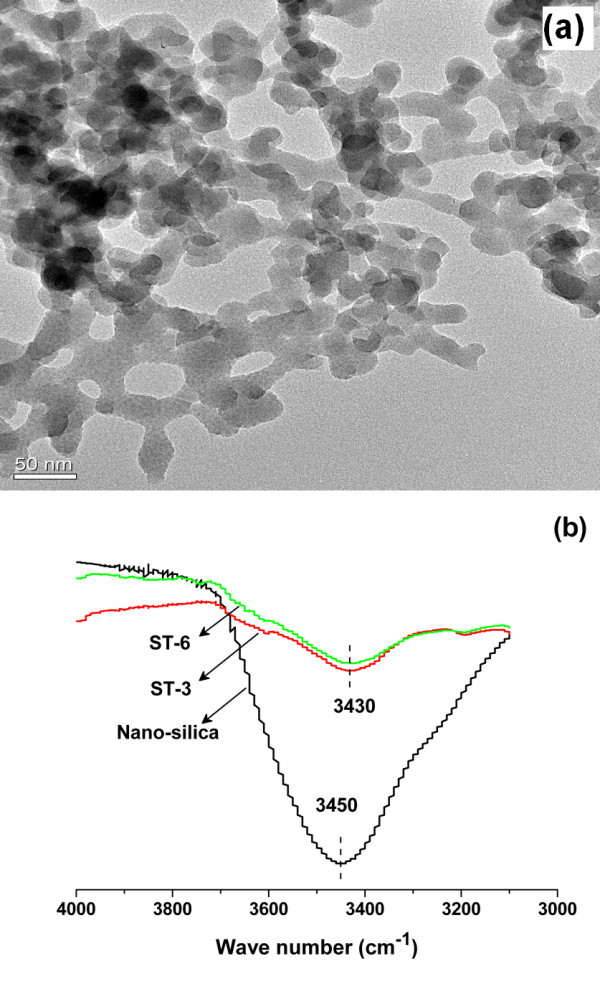
**TEM image and infrared absorption spectrum**. TEM image of a fractal in the suspension **(a) **and the infrared absorption spectrum for the pure nano-silica and the suspensions **(b)**.

The nanoparticle employed in this work is fumed nano-silica which is well-known for the abundance of the hydroxy on the surface [11]. It was found that suspensions of fumed nano-silica in tetradecane became to be a gel when the concentration of the nanoparticle was higher than 3 wt.% owing to the effect of the hydrogen bonds. The diameter of the nanoparticle is 30 ± 10 nm provided by the supplier and confirmed by the TEM images (Figure 1a). At the same time, from Figure 1a, it can be found that the primary nano-silica particles aggregated into short NCA and constructed a gel network in the suspension of nano-silica/tetradecane. Therefore, the aggregates or agglomerates in this study refer to the gel network or NCA. The content of the hydroxy on the surface was determined by acid-basic titration (for details, see additional file 1). By this method, the number of the hydroxy per square nanometer was determined to be about 4. To confirm the existence of hydrogen bonds in gels, the infrared absorption spectrum of pure nano-silica and its suspensions were investigated (Figure 1b). Since free silanol produces a remarkable absorption peak around 3,700 cm^-1 ^and the shift of the peak to lower wave number can be related to the existence of the hydrogen bonds [11], the peaks at 3,450 and 3,430 cm^-1 ^for the pure nano-silica and the gels respectively confirm the existence of the hydrogen bonds.

For the preparation of nano-silica/tetradecane suspensions, the nanoparticle was firstly dried at 120°C for 12 hs to remove the water adsorbed by the nano-silica. To make the gel network structure more perfect, suspensions were dispersed by ultrasonic treatment for 2 h. Five suspensions with weight fraction (wt.%) of nano-silica from 3 to 7 wt.%, named as ST-3, ST-4, ST-5, ST-6 and ST-7, respectively, were prepared at the same conditions. For the preparation of pure nano-silica disks, the same dried nano-silica (ca. 1 g) was first precompressed in the mold (a hollow column with inner diameter of 25 mm) and then compressed under the pressure of 5 MPa for 5 min at the room temperature. Finally, we obtained a disk with a diameter of 25 mm and about 0.7 mm in height for the rheological tests.

### Measurements

Rheological tests were carried out by a stress-controlled rotational rheometer (AR2000EX, TA instruments, USA) with parallel plates (25 mm in diameter) and at the room temperature 25°C. Because the gel network is very weak, in the process of sample loading and rheological measurements, carefulness and some measures are required to keep the gels intact.

For the gel sample loading, we adopted two measures to reduce the unavoidable destroying of the gels. First, the sample was sucked up carefully and slowly from a plastic tube by a pipette which can accurately control the sample volume (we chose a sample volume of 0.420 ml in our work). Secondly, the speed and the force of the compression process to produce an appropriate gap (0.650 mm) for the rheological tests were strictly controlled by the rheometer. For the rheological tests, we first carried out a strain sweep to determine the upper limit srain (ca. 4%) to keep the gels intact and finally chose a strain of 0.5% to perform the frequency sweep and time sweep. Under these measures, the experimental data were found to be repeatable.

## Results and discussion

### Dynamic rheological and mechanical properties

**Figure 2:**
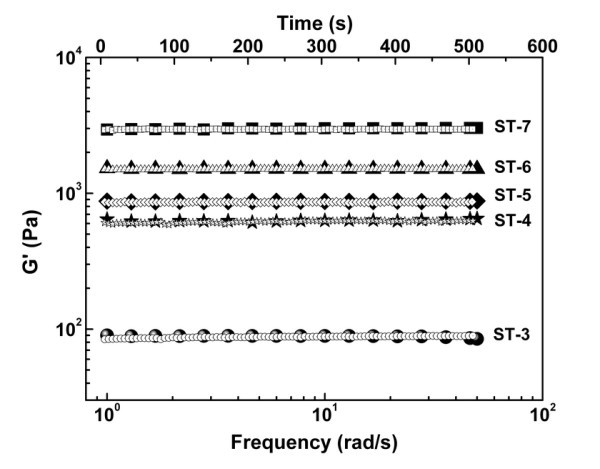
**Dynamic storage modulus (*solid*) and stability (*open*) of the nano-silica/tetradecane gels**.

It is well known that rheology has been a powerful tool to investigate the structures or the structural evolution in materials. As shown in Figure 2 the frequency-independent behavior for the storage modulus (*G'*) is a signature of some elastic network structures [17,18]. Analogous to the network structure of a rubber, the storage or elastic modulus of the gel can be equal to the plateau modulus of the rubber

(1) $$G_{N}^{0}=\rho RT/M_{e}$$

Where, *ρ *the density of rubber, *R *the gas constant, *T *the absolute temperature, *M_e _*the molecular weight of the network strand [19]. It is expected that the storage modulus of the gel depends on the length of the network mesh, i.e., NCA. Therefore, we can establish a relationship between the storage modulus and the length of NCA as follows. First, we assume that NCA has a simple necklace-like shape and can be regarded as an entanglement strand with a molecular weight of *M_e_*. One can easily deduce the relationship between *M_e _*and the length of NCA (*L*_NCA_) as

(2) $$M_{e}=(L_{\text{NCA}}/d_{0})[\rho (\pi d_{0}^{3}/6)]N_{A}=(\rho \pi N_{A}d_{0}^{2}/6)L_{\text{NCA}}$$

Where, *d_0 _*the diameter of nano-silica, *N_A _*the Avogadro constant. Furthermore, we can relate the storage modulus to the length of NCA by

(3) $$G^{\prime }=G_{N}^{0}=\beta \rho RT/M_{e}=6\beta RT/\pi N_{A}d_{0}^{2}L_{\text{NCA}}$$

where *β *is a correction factor to consider the difference in the structure between NCA and polymer chain. It is noted that the structure of NCA may change with the concentration of nanoparticles and make *β *not a constant. For example, a few NCAs may merge into one thick network strand. In this situation, the storage modulus may be different but the length of NCA may be unaltered.

In addition, as also shown in Figure 2 the stability of the gel networks is very conspicuous. For most suspensions of nanoparticles, agglomeration and sedimentation of the nanoparticles are unavoidable and the suspension is commonly unstable [20,21]. Therefore, it can be concluded that the gel network built by the hydrogen bonds can constantly block the agglomeration process as long as the initial agglomerates have been broken apart. This finding may provide an effective approach to improve or stabilize the dispersion of nano-fillers by introducing some additional interaction among the nano-fillers.

**Figure 3:**
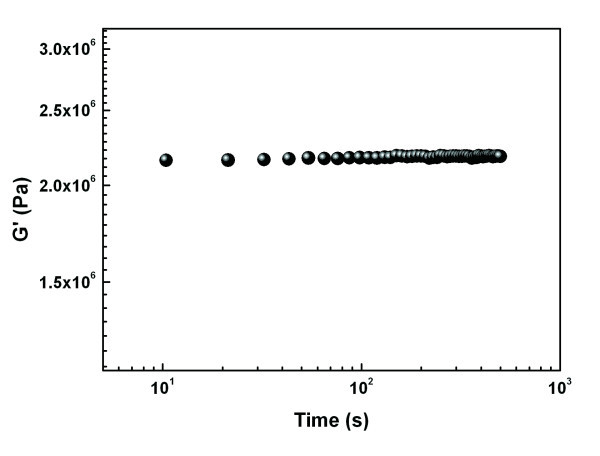
**Dynamic elastic modulus of the nano-silica agglomerate prepared by compression molding at 5 MPa**.

According to the percolation theory [20,22], the relationship between the storage modulus and the concentration *ϕ *(wt.%) can be express by *G' *= *G_s_ϕ^α ^*(*ϕ *≥ *ϕ_c_*, *ϕ_c_*, the critical concentration for the forming of gel network). The power *α *was found to be 3.83 in our study as shown in Figure 3b. It is noted that *G_s _*should have a physical meaning and, here, we propose it as the shear modulus of the agglomerates at the dense packing state (DPS), i.e., the state of *ϕ *= 1, and call it the stack shear modulus. We believe that *G_s _*is a fundamental parameter relating to mechanical properties of agglomerate and is different from that of the bulk. It may be affected by the size, the surface characteristics, and the bulk properties of the nanoparticles. Actually, the agglomerates of nanoparticles can be viewed as a state of quasi-DPS and they are prevalent in nanocomposites. Therefore, this parameter is very important for the nanocomposites when the mechanical properties are of interest. However, as far as we know, the modulus of the bulk, not the aggregates, is usually used to evaluate the contribution of the nanoparticles to the mechanical properties of the nanocomposite [23]. At the same time, the mechanical properties of the agglomerates have been seldom reported [10,24]. For nano-silica employed in this study, we obtain *G_s _*≈ 10^7.92 ^Pa which is obviously lower than the bulk (ca. 10^11 ^Pa) [1], but larger than the experimental result 2.2 × 10^6 ^Pa (Figure 3), which is likely to be caused by the difficulty in compressing the nanoparticles into a disk of DPS on the whole and should be further investigated in the future.

**Figure 4:**
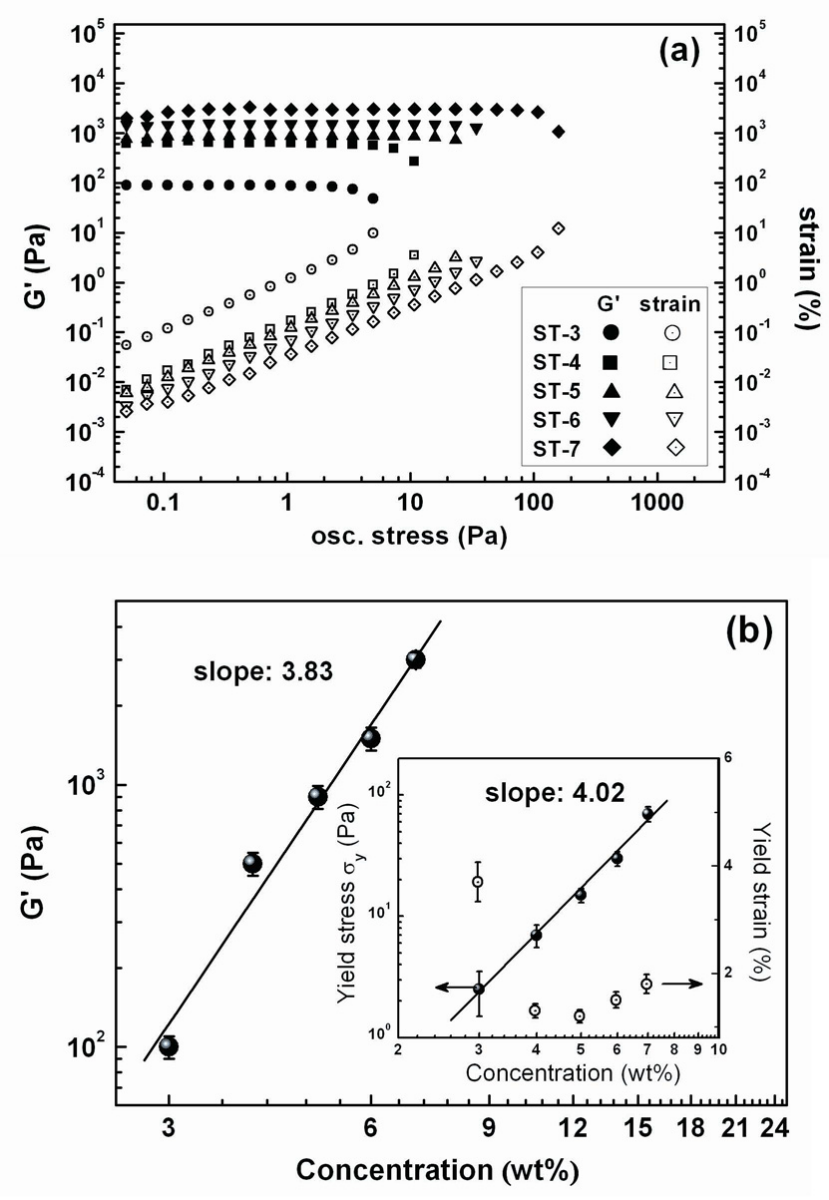
**Sress sweep test and concentration dependence behaviors**. Yielding behavior of the suspensions investigated by stress sweep **(a) **and concentration dependences of the elastic modulus **(b)**. The yield stress and the yield strain is also shown in the inset in *(b)*.

The stress to destroy the gel network (called as the yield stress), and the strain below which the gel can keep intact (called as the yield strain) are the essential mechanical parameters of a gel network, i.e., the aggregates in this study. Stress sweep test carried out by stress-controlled rheometer is very suitable to measure these two parameters at the same time as shown in Figure 4a. It can be found that the elastic modulus is independent on the oscillation stress (σ) and the strain is propotional to σ when the structure is intact. Nevertheless, the stain will increase sharply with the increasing of σ when the structure yield, i.e., the network structure is destroyed. The inset in Figure 4b shows the concentration dependences of the stress and strain at the yield point. It was found that the yield stress *σ_y _*depended on the concentration *ϕ *as $\sigma_{y}=\sigma_{y}^{S}\phi^{4.02}$(*ϕ *≥ *ϕ_c_*), $\sigma_{y}^{S}$ proposed as the yield stress of the agglomerates of DPS and equals to 10^6.5 ^Pa as determined by fitting the experimental data (the inset in Figure 4b). Unfortunately, we were unable to obtain the experimental value of this parameter for the applied stress limitation of the apparatus. But it is obvious that this parameter is significant for the dispersion dynamics of the agglomerates. The exponent (*m *= 4.02) can be related to the fractal dimension (*D_f_*) by equation

(4) $$m=(d+X)/(d-D_{f})$$

where *d *is the Euclidean dimension and equals 3, *X *is the fractal dimension of the backbone of the clusters (i.e., NCA) and it usually takes the value of unity [25]. For the gels investigated here, we have *D_f _*= 2.0, lager than 1.78 [20,21]. This finding indicates that the hydrogen bonds make the fractal more compact [20,26].

The yield strain as revealed in the inset in Figure 4b reflects the extent for elastic deformation of NCA which mainly relates to the length of NCA and the size of the primary nanoparticles [3,6]. It is obvious that, for the suspensions in this study, the length of NCA is too short to generate remarkable elastic deformation. This point is embodied by two aspects: (1) The yield strains of all gels are very small (< 5%). (2) The yield strain, on the whole, seems to decrease with the concentration of the nano-silica increasing. For the second finding, it indicates that the mesh of the network becomes shorter as the concentration of the nano-silica increases. However, it was also found that the yield strains of some high-concentration samples seem to rebound, which may be related to a stronger reconstructability of the gel.

### Rheological properties under continous shear flow

In practice, such as coating and printing, the suspension is inevitable to experience large deformation or continous shearing. In fact, the flow behaviors of all kinds of suspensions (nano- or micro-fillers with different shapes) are always of great interest in the realm of rheology and shear-thinning and shear-thickening behaviors are not unusual [27-32]. However, the flow behavior of the suspensions here is not simple.

**Figure 5:**
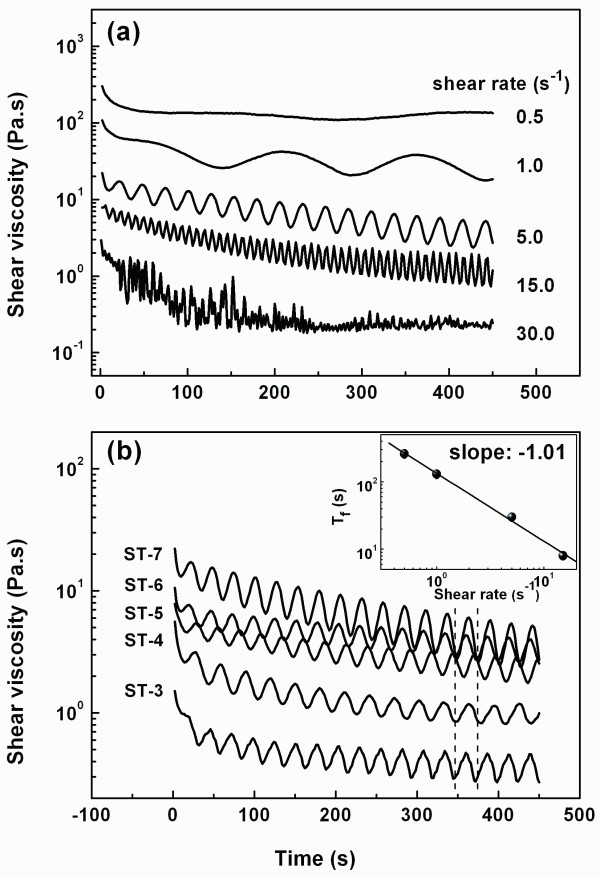
**Shear-thinning behavior**. Viscosity development at different shear rate for the sample ST-7 **(a)**. Viscosity development curves of all samples at the same shear rate of 5 s^-1 ^**(b)**. The inset shows the shear rate dependence of *T_f_*.

Firstly, a shear-thinning behavior, i.e., the shear viscosity declines with the shear rate increase, was observed for all suspensions as displayed in Figure 5a, b. In accordance with the yield behavior, this behavior is also caused by the breaking of gel network occurring when the deformation or strain overpasses the elastic deformation the network or NCAs can support. Obviously, the extent of deformation plays a key role in understanding of the shear-thinning behavior.

**Figure 6:**
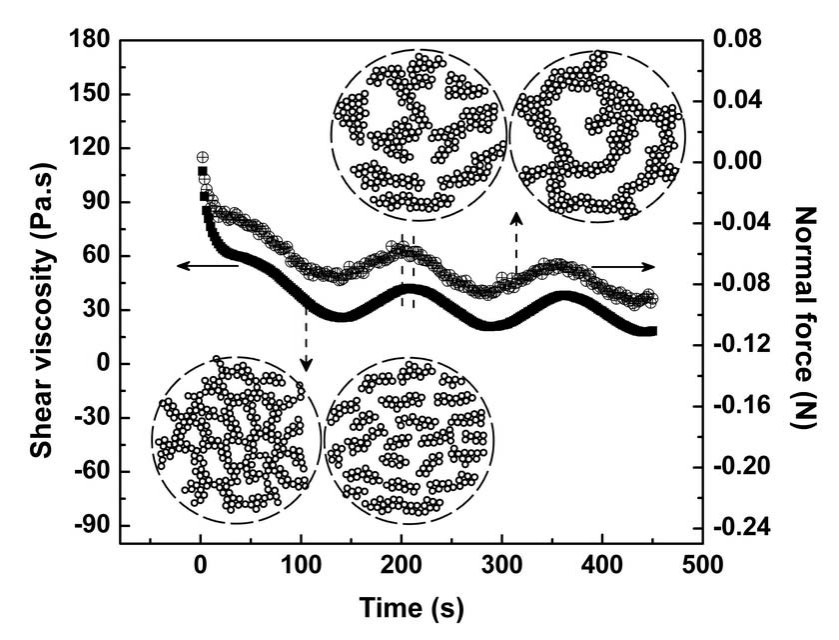
**Destruction and reconstruction of the gel network under steady shear flow of 1 s^-1^**. The change of the structure under shear flow can be detected by the variation of the shear viscosity or the normal force as shown in the plot and discussed in the text.

Secondly, with a constant shear rate, it was observed that the growth curves of viscosity and normal force exhibited periodic fluctuations (Figure 6), indicating a process of destruction and reconstruction of the gel network under continuous shearing flow. This fluctuation behavior can be explained as follows. On the one hand, the breaking of the hydrogen bonds will give rise to a minus normal force because attractive forces mainly coming from the hydrogen bonds will try to rebuild the network. On the other hand, when the reconstruction of the hydrogen bonds exceeds the destruction process, the attractive forces will fade away and result in the upturn of the normal force curve. A schematic of this process is also shown in Figure 6 as denoted by the dashed arrows.

In addition, there are some new characteristics for the continuous shear flow that are worthy to be noted and have been briefly summarized as follows. (1) The periodic time of the fluctuations (*T_f_*) seemed to be only dependent on the shear rate as $T_{f}\sim 1/\dot{\gamma }$ (Figure 5b), in other words, the product of $T_{f}\dot{\gamma }$ is a constant, which once again confirm the key role of deformation in understanding of the rheological properties under continous shear flow. (2) The viscosity declined with time on the whole as the density of the network node descends, which may be related to the mesh thickening that results from the agglomeration of the fractured NCAs or fragments as illustrated by the schematic in Figure 6. (3) It can be found in Figure 6 that there is a retarding behavior between the normal force and the viscosity as indicated by the dashed lines. It is reasonable that the response of the structure (such as the viscosity) is always lagged behind the response of the force (take the normal force for example) because the structure evolution is always the result of the effect of the force.

**Figure 7:**
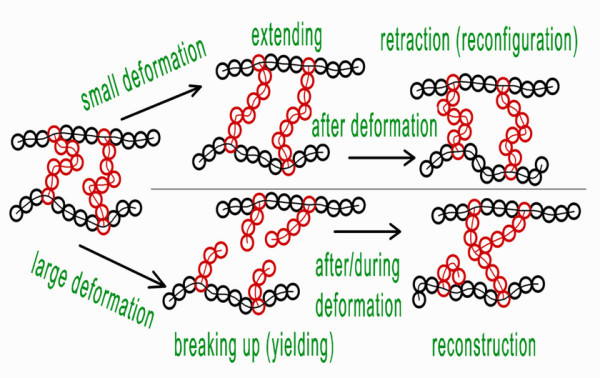
**Schematic configuration/structure changings of the gel network**. Aggregates in the form of NCA can exhibit a polymer-like elastic behavior under small deformation. While under *large deformation*, aggregates will experience *destruction*, *reconstruction*, and *agglomeration *and cannot recover the initial structure.

To help in understanding the rheological and mechanical properties of the gel network or agglomerates, the configuration/structure changings on the nanoscale, such as a NCA, under different deformation conditions are illustrated in Figure 7.

## Conclusion

In summary, the rheological and mechanical properties of nanoparticle agglomerates in the form of network structure have been studied by rheology. Hydrogen bond interaction is found to be a key factor to contribute to the properties of the agglomerates. The elastic network model for rubber can be modified to link the mesh size of the network to the dynamic modulus. Furthermore, by rheology, we can define two important parameters, the stack shear modulus and the yield stress of the agglomerate at the DPS, which may be very valuable in nano-science. Under continous shear flow, the structure of the aggregates experiences some repeating process of destruction, reconstruction and agglomeration.

## Competing interests

The authors declare that they have no competing interests.

## Authors' contributions

YW designed the present work and carried out most of the experimental work (including the preparation and characterizations of the samples and the rheological tests) and the analysis of experimental data XJW participated in most of the experimental work and the analysis of experimental data. WY conceived of this study and directed the preparation of the manuscript. YMZ participated in the discussion of this work. Prof. BH Xie and Prof. MB Yang offered physical and spiritual supports to this work. All authors read and approved the final manuscript.

## Supplementary Material

Additional file 1 **Characterizations of the materials and additional rheological properties**. It contains the specifics of the characterizations of the materials, schematic of the network forming under ultrasonic treatment and additional figures for the rheologcial properties of the aggregates. Click here for file
