# Supplementary material for: Aggregate of nanoparticles: rheological and mechanical properties
Source: Nanoscale Res Lett. 2011 Feb 3;6(1):114. doi: 10.1186/1556-276X-6-114 (PMC3211159; doi:10.1186/1556-276X-6-114)
Supplement: Additional file 1 — Characterizations of the materials and additional rheological properties. It contains the specifics of the characterizations of the materials, schematic of the network forming under ultrasonic treatment and additional figures for the rheologcial properties of the aggregates. [file 1556-276X-6-114-S1.DOC]

**Electronic Supplementary Material for:**

**Agglomerate of Nanoparticles: Rheological and Mechanical Properties**

Yu Wang, Xiao-Jun Wu, Wei Yang*[[1]](#footnote-2)**, Yuan-Ming Zhai, Bang-Hu Xie, Ming-Bo Yang

College of Polymer Science and Engineering, State Key Laboratory of Polymer Materials Engineering, Sichuan University, Chengdu, 610065, China

[**1. Details for the determination of the content of the hydroxy on the nano-silica surface 1**](#__RefHeading___Toc270196538)

[**2. Figures 2**](#__RefHeading___Toc270196539)

[**Fig. S1. 2**](#__RefHeading___Toc270196540)

[**Fig. S2. 3**](#__RefHeading___Toc270196541)

[**Fig. S3. 4**](#__RefHeading___Toc270196542)

[**Fig. S4. 5**](#__RefHeading___Toc270196543)

[**Fig. S5. 6**](#__RefHeading___Toc270196544)

# 1. Details for the determination of the content of the hydroxy on the nano-silica surface

The content of the hydroxy on the surface was determined by acid-basic titration. The specific operation is summaried as follows. First, the dried fumed nano-silica (2.01 g) was dispersed with the mixture of ethanol (25 ml) and NaCl solution (75 ml, 20 wt%) in a beaker (200 ml). To help the dispersion, magnetic stirring was constantly used in the whole measurement. After stirring of about half an hour, the pH of the suspension was tuned to be around 4 by the addition of HCl or NaOH solution (0.1 mol L^-1^). Then, the pH was tuned to be around 9 by dripping slowly NaOH (0.1 mol L^-1^) solution and hold this value for 30 s. By the dosage of NaOH solution to tune the pH from 4 to 9, we can estimate the number (*N*) of the hydroxy per nm^2^ by

*N* = *CVN_A_*×10*^-3^ / Sm*

where *C* the concentration of NaOH solution, mol L^-1^, V the wastage of NaOH solution, ml, *N_A_* the Avogadro constant, *S* the specific area of the nanoparticle, nm^2^ g^-1^, *m* the mass of nanoparticles, g.

# 2. Figures


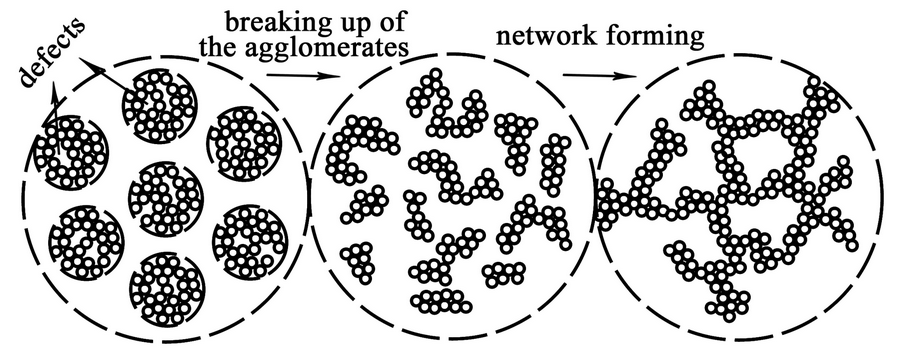

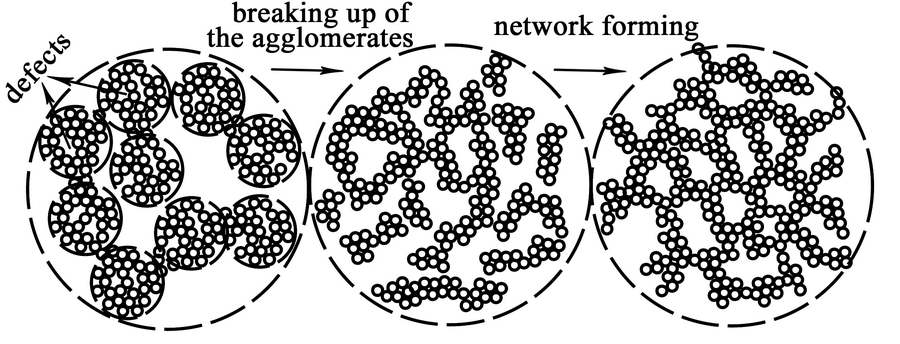


Fig. S1. Schematic of the network forming (top) or perfection (bottom) in the suspension of nano-silica/tetradecane under the ultrasonic treatment. For low concentration suspension (ST-3), it was found that the gel network on the whole can not form unless it was treated by ultrasonic and this situation is pictured by the top one. For high concentration suspensions (ST-4, ST-5, ST-6, ST-7), the perfection of the gel network may be the main event in the process of ultrasonic treatment as shown in the bottom one.

**
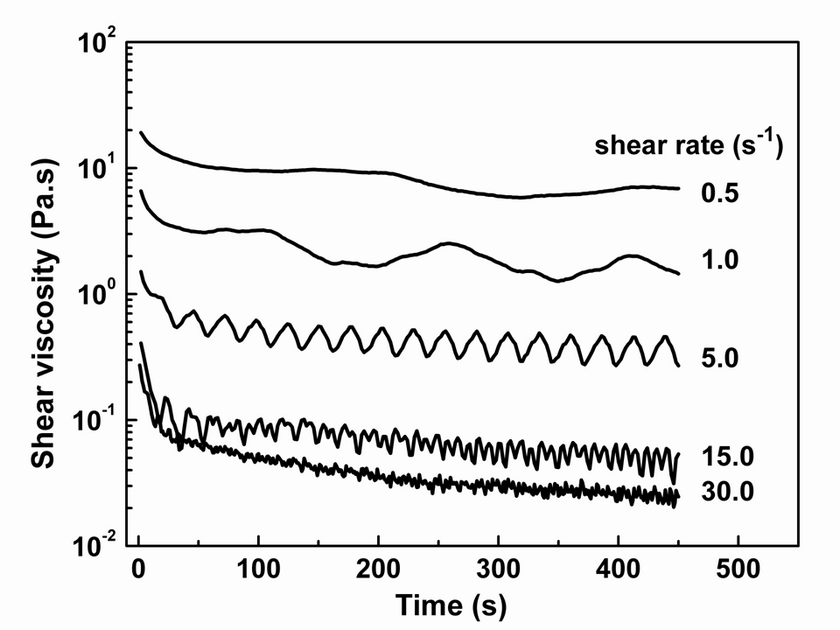
**

Fig. S2. Viscosity development at different shear rate for the sample ST-3.

**
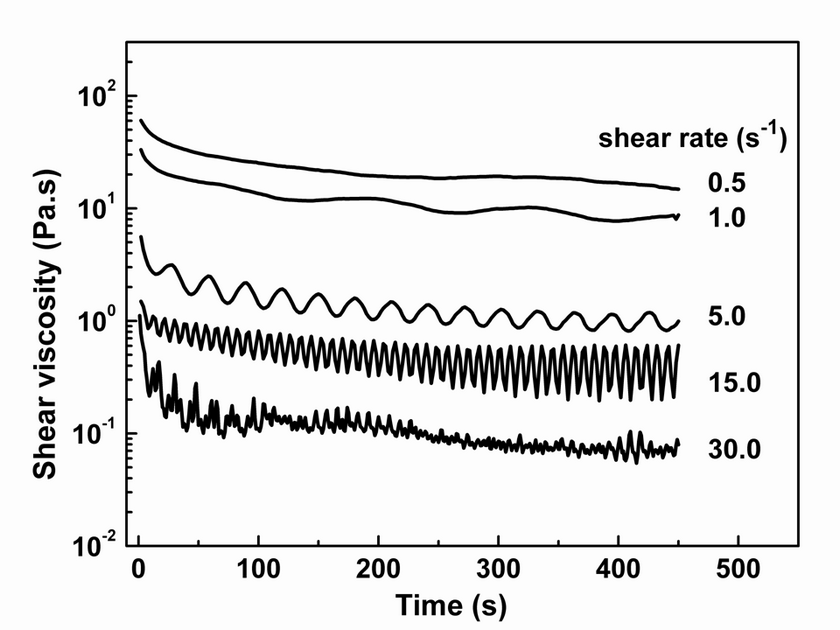
**

Fig. S3. Viscosity development at different shear rate for the sample ST-4.


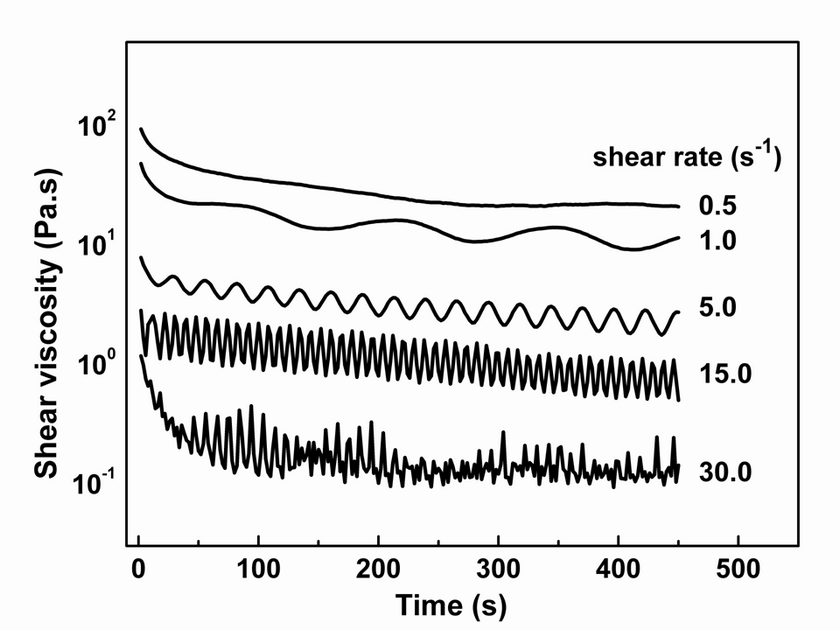


Fig. S4. Viscosity development at different shear rate for the sample ST-5.


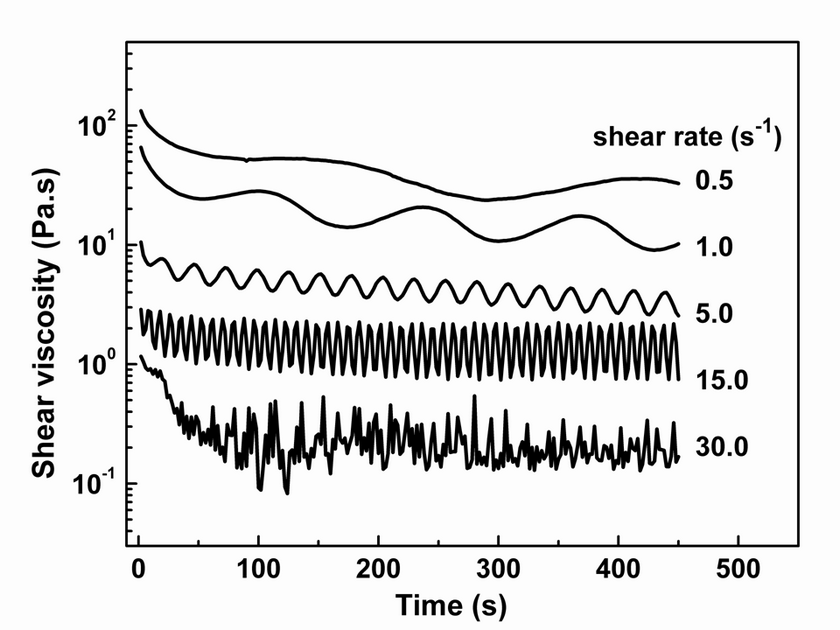


Fig. S5. Viscosity development at different shear rate for the sample ST-6.

1. ^*^ Corresponding author, Email: weiyang@scu.edu.cn [↑](#footnote-ref-2)
